# Androgen Receptor Mutations Associated with Androgen Insensitivity Syndrome: A High Content Analysis Approach Leading to Personalized Medicine

**DOI:** 10.1371/journal.pone.0008179

**Published:** 2009-12-09

**Authors:** Adam T. Szafran, Sean Hartig, Huiying Sun, Ivan P. Uray, Maria Szwarc, Yuqing Shen, Sanjay N. Mediwala, Jennifer Bell, Michael J. McPhaul, Michael A. Mancini, Marco Marcelli

**Affiliations:** 1 Department of Molecular and Cellular Biology and Medicine, Baylor College of Medicine, Houston, Texas, United States of America; 2 Michael E. DeBakey VA Medical Center and Department of Medicine, Baylor College of Medicine, Houston, Texas, United States of America; 3 Department of Pediatrics, Baylor College of Medicine, Houston, Texas, United States of America; 4 Department of Internal Medicine, Division of Endocrinology and Metabolism, The University of Texas Southwestern Medical School, Dallas, Texas, United States of America; Brunel University, United Kingdom

## Abstract

Androgen insensitivity syndrome (AIS) is a rare disease associated with inactivating mutations of AR that disrupt male sexual differentiation, and cause a spectrum of phenotypic abnormalities having as a common denominator loss of reproductive viability. No established treatment exists for these conditions, however there are sporadic reports of patients (or recapitulated mutations in cell lines) that respond to administration of supraphysiologic doses (or pulses) of testosterone or synthetic ligands. Here, we utilize a novel high content analysis (HCA) approach to study AR function at the single cell level in genital skin fibroblasts (GSF). We discuss in detail findings in GSF from three historical patients with AIS, which include identification of novel mechanisms of AR malfunction, and the potential ability to utilize HCA for personalized treatment of patients affected by this condition.

## Introduction

Androgen action is mediated by the intracellular androgen receptor (AR), a transcription factor member of the nuclear receptor superfamily. While in the cytoplasm under baseline conditions, upon addition of agonist AR translocates to the nucleus, where it interacts with coregulators and promoters/enhancers of AR-responsive genes, and regulates transcription. AR action is a *conditio sine qua non* for the normal development and function of the entire male genital tract; conversely, varyingly degrees of impaired AR action from mutation is causative in individuals affected by androgen insensitivity syndrome (AIS) [1], [2]. Three main clinical phenotypes in humans define AIS: Complete, Partial and Minimal Androgen Insensitivity (CAIS, PAIS and MAIS), and they range from complete lack of virilization of the internal and external genitalia (CAIS), to intermediate virilization (PAIS), to apparently normal virilization in infertile males (MAIS) [1]. A slightly more complex classification, describing seven grades of abnormal virilization, has been proposed by Quigley and collaborators [2]. A data-base of AR mutations in AIS patients is published on-line (http://androgendb.mcgill.ca/), and a large body of previous work has defined three broad varieties of AR ^3^H-DHT binding abnormalities in monolayer binding analyses: **1)** absent binding (i.e. ^3^H-DHT binding is undetectable) [3]; **2)** qualitatively abnormal binding [e.g., binding is normal but with qualitative abnormalities such as increased ligand dissociation rate (the dissociation rate is considered abnormal if <60% of the specific androgen binding remains after 3 hours) [4]; or thermolability (defined as a reduction in specific androgen binding at 41°C compared to 37°C of greater than 40%) [5]]; or, **3)** decreased binding (e.g., binding is detectable but below normal) [1]. The degree of abnormality caused by each individual mutation is usually related to the patient phenotype, the ^3^H-DHT binding characteristics, and the amount of residual reporter gene activity present in cells transfected with an AR carrying that particular mutation; in general, in the more feminized phenotypes, lack of ^3^H-DHT binding and abnormal transcriptional activity parallel increasing AR malfunction.

Despite the clinical dogma that “AIS is not treatable,” some sporadic PAIS and MAIS patients respond to endocrine management consisting of pharmacologic doses of androgens [6]–[9]. Further, in vitro analysis of some AR mutations with the binding phenotype of normal ^3^H-DHT dissociation constant (K*_d_*) and maximal binding (B_max_), but increased ligand-receptor dissociation rate can be normalized under certain culture conditions. For instance, such an AR containing a single amino acid substitution (Y763C) and a reduced polyglutamine tract (Q12) normalized its transcriptional activity when exposed to pharmacologic concentrations of androgens both in vitro [10] and in vivo [6]. In other such CAIS or PAIS mutations, transcriptional activity normalized either after administration of supraphysiologic concentrations of endogenous androgens (e.g., testosterone or dihydrotestosterone, DHT), synthetic androgens (Mibolerone or R1881), or after treatment with frequent pulses (up to every four hours) of physiologic doses of DHT [11]–[13].

Our group has recently developed a high throughput microscopy-based technology to simultaneously analyze multiple AR activities at the single cell level, an approach often referred to as high content analysis (HCA). Our AR-oriented HCA involves multi-parametric interrogation of cultured cells using automated high magnification, high resolution imaging and immunofluorescence or green fluorescent protein-fused AR (GFP-AR) in combination with use of a red fluorescent protein-based transcriptional reporter protein [14]–[17]. Utilizing custom-developed image analysis routines, the datasets can be quantitatively explored to yield a “multiplex” view of interrelated AR functions. The image data mining yields information including (but not limited to) AR expression, subcellular trafficking, reporter gene activity, cell cycle position, mitotic index, and literally hundreds of other measurements per cell [15].

We theorized that HCA of AR would be an ideal technology to directly analyze patient-derived genital skin fibroblasts to identify not only mechanisms associated with abnormal AR activities, but also therapeutic options normalizing mutant AR functions. While there are many AIS mutations described, those localized in the LBD and associated with 1) normal K*_d_*, 2) normal B_max_, and 3) qualitative abnormalities of ligand-receptor interaction would be the most amenable to normalization. We present here unique cytological profiles generated by HCA from three historical patients affected by CAIS or PAIS, which were complemented by NH_2_-COOH-terminal domain interaction (NC-TDI) experiments, and previously published ligand-binding studies [11]. All patients carried AR mutations with the specifications listed above. HCA revealed the type of functional defects associated with these mutations, and ligand-dependent restoration of AR functions using experimental conditions that increase the stability of the ligand receptor complex in two of the three patients. Normalization of AR function was associated in each case with improvement of NC-TDI. These studies provide a proof-of-concept demonstration that specific clinical mutations of AR can be examined by HCA for use in personalized medicine.

## Methods

### Cell Culture

Primary cultures of genital skin fibroblast from AIS patients F764L, R840C and P766S, and six normal patients were established as previously described [18], and maintained in MEM with 10%FBS and 1% penicillin and streptomycin. HeLa cells were obtained from the American Type Culture Collection (Rockville, MD) and were maintained in DMEM supplemented with 5%FBS and 1% penicillin and streptomycin. Control GSF cell lines were isolated at the time of circumcision from the foreskin of male neonates with normal genital development, after obtaining approval from the Baylor College of Medicine Institutional Review Board. No further follow up on these individuals is available, and it is assumed that normal pubertal development will be completed, and that their AR sequence is wild type. Patients with PAIS or CAIS were from the historical library of AIS patients at UT Southwestern [1].

### AIS Mutation Background and Clinical History

The mutations investigated in this study are AR-F764L, AR-R840C, and AR-P766S. These mutations were previously identified in patients affected by either PAIS, or CAIS, and functionally characterized by using either patient derived genital skin fibroblasts, or after transfection in CV1 or CHO cells [11]. The discrepancies between the coordinates of the mutations reported in this paper and in the original publication [11] are due to the use in this publication of the AR coordinates reported in the AR mutation database online (http://androgendb.mcgill.ca/). The discrepancies are due to the different number of glutamines in the polyglutamine region of exon 1 that were reported in the initial sequences of AR.

### Mutation F764L

The F764L mutation was originally identified in a CAIS patient of Dr. G. Costin (Los Angeles, CA) [11], and has also been reported in other patients by two additional groups [19], [20]. Biochemical studies in transfected CHO cells demonstrated normal ^3^H-DHT K*_d_* and B_max_ (the K*_d_* was 0.26 nM for the wild type AR and 0.1 nM for mutant F764L, which is not considered a meaningful difference), and abnormally elevated ligand dissociation rate [11]. Using DHT and the MMTV-luc reporter, the F764L mutation demonstrated significantly decreased transcriptional activity in transfected CV1 cells. Activity was rescued to nearly 30% of control by the synthetic androgen Mibolerone [11].

### Mutation R840C

This mutation has been identified in multiple patients with PAIS [21]–[26]. Biochemical studies in transfected CHO cells demonstrated normal ^3^H-DHT B_max_ and K*_d_* and presence of thermolability [11]. When examined in transfected CV1 cells, the transcriptional activity of the R840C mutated receptor was decreased to 67% of control using DHT [11].

### Mutations P766S

This mutation was identified in three patients with CAIS [11], [27], [28]. Biochemical characterization in CHO cells revealed normal K*_d_* and B_max_, and abnormally elevated ligand dissociation rate. Transcriptional activity was 9% of control in response to DHT, but normalized at 120% of control in response to Mibolerone [11].

This historical analysis showed that AR-R840C, AR-F764L and AR-P766S shared these following common features; they were point-mutations localized in the ligand binding domain with normal ^3^H-DHT K*_d_* and B_max_. According to the series of 130 families published by Dr. Griffin, these features are shared by 40% of patients with AIS [1].

### GFP-AR Expression Vectors and HeLa Stable Cell Lines

cDNA expression vectors for hAR harboring the AIS related mutations were previously generated [11]. Generation of pEGFP-AR fusion vectors with an NH_2_-terminal GFP tag and regulation by the CMV promoter has been previously described [29]. pEGFP vectors expressing AR harboring the various AIS mutations were generated by replacing an EcoRI-Pvul cDNA fragment encoding the LBD of pEGFP-AR by the same fragment of pAR-F764L, pAR-R840C, pAR-P766S expression vectors.

A HeLa cell line stably expressing wild type GFP-AR was previously described [15]. To generate similar cell lines expressing the various AIS associated mutations, HeLa cells were transfected with 1 µg/well pEGFP-AR plasmid DNA using BioRad Transfectin reagent 1 day after plating in six-well plates. After 24 hours, cells were trypsinized and plated in medium supplemented with 1 mg/mL G418 (Invitrogen) in 10 cm tissue culture dishes. Clones were selected, expanded, and analyzed by flow cytometry for GFP-AR expression. Cells with low to moderate GFP-AR expression were then single cell cloned and expression verified by western blot; subsequently, GFP-AR expression was found similar to or below the endogenous expression of AR in the LNCaP prostate cancer cell line [15].

### NH_2_-COOH-Terminal Domain Interaction (NC-TDI) Assay

An interaction between COOH terminal AF2 (activation function 2) and NH_2_ terminal sequence F^23^XXLF^27^ is an important step toward the activation of AR [30]. The NC-TDI was measured using the reagents of the CheckMate™/Flexi® Vector Mammalian Two-Hybrid System, Promega (Madison, WI). A segment containing AR 1-660 was fused to the VP16 TAD domain of plasmid pFN10A, while segments AR 624-919 (wild type or containing mutations F764L, R840C, and P766S) were fused to the to the Gal4-DBD domain of plasmid pFN11A. The NC-TDI assay was performed in a similar way as a standard transactivation assay, except for the expression vectors that were used. One day before transfection, 1.5×10^6^ Hela cells were seeded into a 60 mm dish. Cells were co-transfected with 1.3 ug of pFN11A-AR624-919, 1.3 ug of pFN10A-1-660, 1.3 ug of pGL4.31 (containing five GAL4 binding sites upstream of a minimal TATA box, which is upstream of a firefly luciferase gene that acts as a reporter for interactions between proteins), and 1 ng of phRL-TK carrying the Renilla luciferase gene. After 12 hours of transfection, cells were trypsinized and equally seeded into nine wells of a 12 well plate, with one well treated with vehicle and others with various concentration of different ligand. After 24 h of treatment, luciferase activity was assayed with the Promega Dual Glo assay kit, using a luminometer (PerkinElmer). Data represent the average±SD of three independent experiments, are expressed as fold-induction compared with negative control, and represent the units of firefly luciferase corrected for the units of renilla luciferase detected in the same plate.

### Preparation of Cell Lines and Experimental Procedure

To determine the global effect of the various AIS mutations on AR signaling, each was analyzed using HCA. This technology allows simultaneous quantification of AR protein expression, nuclear translocation, subnuclear patterning, and transcriptional activity [15]. The analysis was done in HeLa cell lines stably transfected with wild type or mutant ARs fused with green fluorescent protein, or using AR immunofluorescence in primary cultures of genital skin fibroblasts generated from foreskin biopsies of historical AIS patients, or of normal neonates undergoing circumcision at birth. To prevent the effects of receptor over-expression [15], stable HeLa cells were carefully selected to express AR levels similar to LNCaP as previously described [15]. Prior to ligand treatment, HeLa cells were transfected with the pARR_2_PB-dsRED2skl reporter plasmid, consisting of the AR responsive promoter ARR_2_PB [31] driving expression of the red fluorescent protein reporter dsRED2skl engineered to target peroxisomes [15]. Stably transfected HeLa cells, containing wild type or mutant ARs, and GSF cell lines were treated with DHT, R1881 and Mb at concentrations ranging from 100 to 0.001 nM for 24 hours.

### High Throughput Microscopy – Sample Preparation

For studies utilizing HeLa GFP-AR cell lines, twenty-four hours before transfection with pARR_2_PB-dsRED2skl, cells were plated onto 100 mm plastic dishes in medium supplemented with 5% charcoal-stripped and dialyzed-FBS. Transient introduction of the pARR-2PB-dsRED2skl reporter construct was performed using 6.0 µg reporter plasmid and 6.0 µg carrier DNA (BlueScript, Stratagene, San Diego, CA) using Transfectin (Biorad) following standard protocols. After 8-hour incubation, DNA/lipid complexes were removed. Cells were then trypsinized and replated at 10,000 cells per well in Nunc poly-D-lysine treated 96-well optical glass bottom plates and incubated an additional 12 hours to allow for cell adhesion. Cells were then exposed to DHT, R1881 or Mibolerone for 24 h. Compound dilutions and final addition to multi-well plates were performed using a Beckman Biomek NX robotic platform to ensure repeatability from experiment to experiment. After incubation was complete, using the Biomek NX robot, plates were washed with PBS and fixed for 20 min at RT in 4% formaldehyde prepared in CSK buffer (80 mM potassium PIPES, pH 6.8, 5 mM EGTA, 2 mM MgCl2). After fixation, cells were briefly permeabilized (5 min) with 0.5% Triton-X and prepared for imaging by washing in PBS, aspirating the washed solution, and adding a 1 ng/ml DAPI solution to stain DNA and a generic protein stain (CellMask, Invitrogen) to facilitate cell and nuclear image segmentation. Cells were imaged in PBS.

For studies utilizing GSF cell lines, cells were treated in an identical manner except cells were not transfected with the reporter construct. After ligand incubation was complete, cells were fixed and endogenous AR was immunolabeled using the Biomek NX liquid handling robot. Cells were initially washed in PBS buffer and fixed for 30 min on ice in 4% formaldehyde prepared in CSK buffer. After fixation, auto-fluorescence was quenched using a 0.1 M NH_4_Cl solution for 10 minutes. Next, cells were permeabilized (30 min) with 0.5% Triton-X. After washing, cells were incubated for 30 minutes in 5% non-fat milk in Tris buffered Saline (TBS)-Tween (Blotto) followed by an overnight incubation in Blotto containing a 1 µg/ml anti-AR mouse monoclonal antibody (AR ms IgG [32], a kind gift from Dr. Dean Edwards, Baylor College of Medicine). The primary antibody was labeled using an anti-ms IgG Alexa488 secondary, fixed and prepared for imaging by washing in PBS, aspirated and then washed, then adding a 1 ng/ml DAPI/CellMask solution. Cells were imaged in PBS.

### High Throughput Microscopy – Image Acquisition

Cells were imaged using the Cell Lab IC-100 Image Cytometer (IC-100; Beckman Coulter) platform that consists of 1) Nikon Eclipse TE2000-U Inverted Microscope (Nikon; Melville, NY); 2) Chroma 82000 triple band filter set (Chroma; Brattleboro, VT); 3) an imaging camera: Hamamatsu ORCA-ER Digital CCD camera (Hamamatsu; Bridgewater, NJ); and 4) a focusing camera: Photonics COHU Progressive scan camera (Photonics; Oxford, MA). The microscope was equipped with a Nikon S Fluor 40X/0.90NA objective and the imaging camera set to capture 8 bit images at 2×2 binning (672×512 pixels; 26.0 µm^2^ pixel size) with 4 images captured per field (DAPI, GFP/A488, dsRED2skl, A647). In general, 49 to 64 images were captured per well for image analysis.

### High Throughput Microscopy – Image Analysis

**Figure 1:**
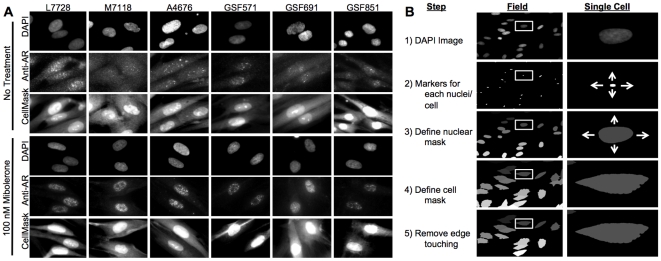
Androgen Receptor High Content Analysis (AR HCA). A, Sample of untreated or treated (with 100 nM Mibolerone) GSF images captured with the IC-100 microscope showing three controls (L7728, M7118, A 4676), and three AIS patients [indicated as GSF strain 571 (AR mutant F764L), 691 (AR mutant R840C) and 851(AR mutant P766S)]. For each experimental condition, the first rows show DAPI, AR, and cell mask staining. B: Pipeline Pilot handling of the fluorescent images, starting from identification of the nucleus, of the cell mask, to computation of the green channel (i.e. AR staining) features under various experimental conditions.

Fig. 1A shows examples of typical images obtained with the IC-100 microscope of normal GSFs (L7728, M7118, A4676) or GSFs from AIS patients [marked as GSF strain 571 (AR mutant F764L), 691 (AR mutant R840C) and 851(AR mutant P766S)], stained with DAPI, AR, and CellMask solution, at concentrations of Mibolerone of 0 and 100 nM. While our initial AR studies using HTM were based upon CytoShop (Beckman-Coulter) [14], [15], [33], these approaches were refined in the current work with the more robust server-client Pipeline Pilot image analysis platform (Basic and Advanced Imagine Collections, Pipeline Pilot 7.5, Accelrys, San Diego). Initially, the background signal was removed from all images using plate- and channel-specific correction images, generated by the sum projection of >600 randomly selected images for each channel from the image set. This allows for a pixel-by-pixel background subtraction that compensates any consistent uneven background artifacts in the image set. Furthermore, users are able to customize the degree of background subtraction by applying a multiplier to each correction image, useful for identification of objects such as nuclei or cell borders. After background subtraction, nuclear masks were generated using a combination of non-linear least squares and watershed-from-markers image manipulations of the DAPI images (Fig. 1B). Cell border masks were generated using the CellMask-labeled images, and watershed-from-markers image manipulations. Due to the spindly shape of the genital skin fibroblasts, a maximal distance from the nucleus limit was placed on the generated cell masks. Cell populations were filtered to achieve uniformity, e.g., without cell aggregates, mitotic cells, apoptotic cells, or cellular debris.

Applied gates were based upon 1) nuclear area; 2) nuclear circularity [(4*π*area)/perimeter^2^]; and, 3) DNA content (DAPI sum of pixel intensities) (Fig. 1B). AR expression was determined by quantifying the sum of pixel intensities in the AR channel within the defined cell region for each cell. AR nuclear hyperspeckling is the statistical variance in AR pixel intensity within the nuclear mask. Accumulation of the AR-sensitive probasin transcriptional reporter was determined by measuring the sum of pixel intensities in the dsRED2skl channel within the cell region for each cell. Finally, the degree of AR nuclear translocation was determined by measuring the percent of total AR signal localized within the nuclear mask. EC50 values were calculated by plotting a simple scatter plot of response versus ligand concentration using the SigmaPlot four parameter logistic curve-fitting algorithm. Due to the nature of the curve-fitting algorithm, for those responses that did not plateau, the response observed at the highest concentration was assumed maximal. Changes in nuclear translocation with ligand treatment are reported as the percentage point change in the percent of the signal in the nucleus compared to untreated samples (i.e. %_Treated_ = 75.1, %_Untreated_ = 35.2, %_Response_ = 39.9). All other responses with ligand treatment are reported at fold change compared to untreated samples (i.e. Reporter_Treated_ = 1000, Reporter_Untreated_ = 100, Reporter_Response_ = 10). Determination of significant differences between compounds was accomplished by first performing an ANOVA analysis followed by a post-hoc multiple comparison analysis with significance set at <0.05.

**Figure 2:**
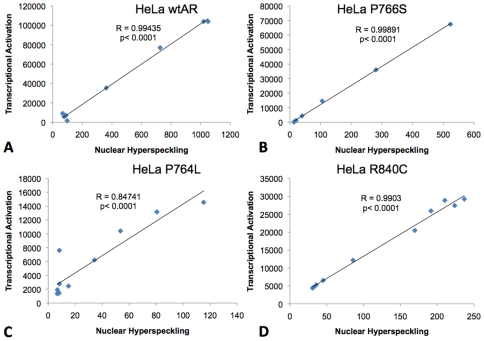
Correlation analysis between nuclear hyperspeckling and transcriptional activity. HeLa cells were stably transfected with wild type AR (A), AR-P766S (B), AR-F764L (C) and AR-R840C (D) and analyzed. Each point of the regression curve was derived from stimulation with logarithmic concentrations of DHT (10^−15^ to 10^−6^ M).

Pearson's correlations analysis was performed to verify the relationship existing between AR nuclear hyperspeckling and transcriptional activation in HeLa cells. For each mutant and ligand used, a strong positive correlation between these two parameters was confirmed, with R^2^ values close to 1, regardless of the ligand or AR construct used. An example of this analysis for HeLa cells stably transfected with wild type AR, AR-F764L, AR-R840C or ARP766S and treated with DHT is shown in Fig. 2. In this experiment, the R^2^ value ranged between 0.85 and 0.999, and *p* values were <0.0001.

## Results

### NC-TDI of AR Mutants

**Figure 3:**
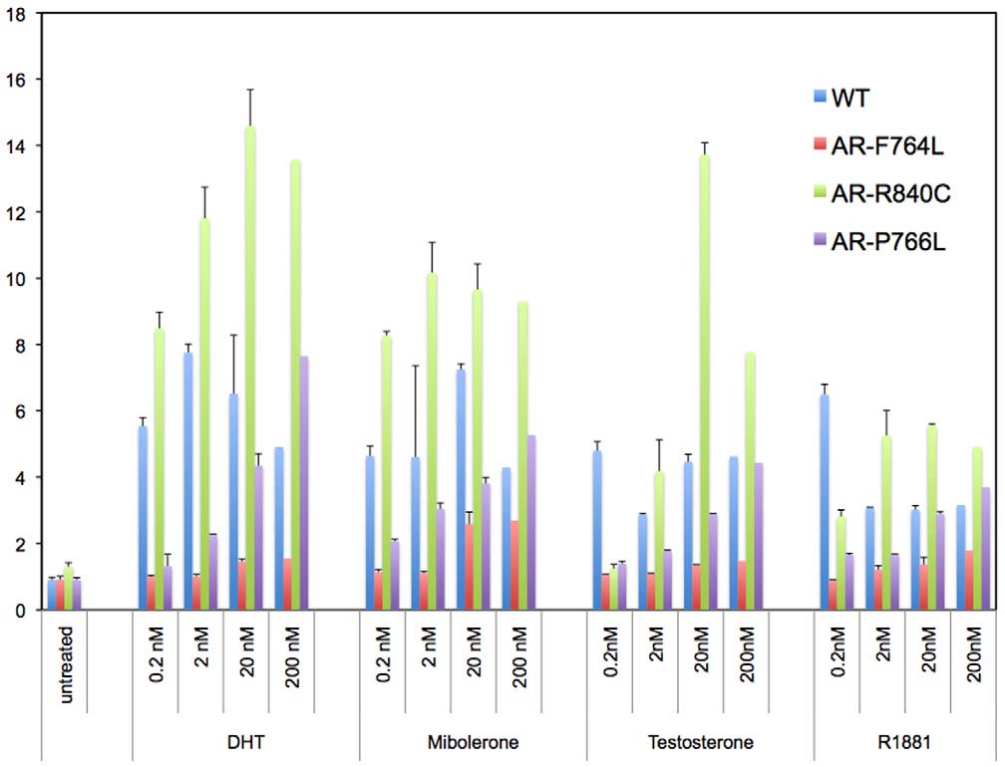
NH_2_-COOH-terminal domain interaction (NC-TDI) assay. A segment containing AR 1-660 was fused to the VP16 TAD domain of plasmid pFN10A CheckMate™/Flexi® Vector Mammalian Two-Hybrid System, Promega (Madison, WI), while segments AR 624-919 (wild type or containing mutations F764L, R840C, and P766S) were fused to the to the Gal4-DBD domain of plasmid pFN11A. One day before transfection, 1.5×10^6^ HeLa cells were seeded into a 60 mm dish. Cells were co-transfected with 1.3 ug of pFN11A-AR624-919, 1.3 ug of pFN10A-1-660, 1.3 ug of pGL4.31 (containing five GAL4 binding sites upstream of a minimal TATA box, which is upstream of a firefly luciferase gene that acts as a reporter for interactions between proteins), and 1 ng of phRL-TK carrying the Renilla luciferase gene. After 12 hours of transfection, cells were trypsinized and equally seeded into nine wells of a 12 well plate, with one well treated with vehicle and others with various concentration of different ligand. After 24 h of treatment, luciferase activity was assayed with the Promega Dual Glo assay kit, using a luminometer (PerkinElmer). Data represent the average±SD of three independent experiments, are expressed as fold-induction c/w negative control, and represent the units of firefly luciferase corrected for the units of renilla luciferase detected in the same plate.

NC-TDI is an important event in the regulation of AR activity [34], and improper regulation of this interaction could be involved in the abnormalities of the AIS mutations examined here. When wild type AR was examined with T, DHT, R1881 and Mibolerone used at 0.2–200 nM, all compounds were able to induce an increase in luciferase activity at the lowest concentration (0.2 nM; Fig. 3). The maximal responses of the tested compounds ranged between a 3.2-fold increase and a 7.7-fold increase (T – 6.4, DHT – 7.7, Mb – 7.1, R1881 – 3.2). While the responses of the weaker AR agonist T were dose-dependent, the three stronger AR agonists (DHT, R1881, and Mb) lacked a dose-dependent response, and reached their plateau at subnanomolar concentrations. **AR-F764L** did not respond to T and DHT, however it responded partially to R1881 (1.8-fold at 200 nM), and maximally (3.1- fold at 200 nM) to Mibolerone. NC-TDI increased from 10% to 50-60% of wild type receptor at 0.2 and 200 nM of Mibolerone, respectively. Thus, increasing the concentration of Mibolerone to suprasaturating concentrations elicited a partial rescue of NC-TDI in mutant **AR- F764L** (Fig. 3). Analysis of **AR-R840C** resulted in significantly larger induction of the NC-TDI response when compared to wild type AR with any of the ligands tested. The response was dose-dependent with T and DHT, while it saturated at 2 nM with the synthetic androgens Mb and R1881 (Fig. 3). In contrast, there was a clear dose-dependent response to all ligands tested for **AR-P766S**. The common denominator of the experiments with AR-P766S was that doses of 200 nM of T, DHT, Mib or R1881 were able to rescue the NC-TDI response to the levels observed with wt AR at concentrations of 0.2 nM (Fig. 3).

### Image Based Analysis of Wild Type AR Functions

#### HeLa cells

**Figure 4:**
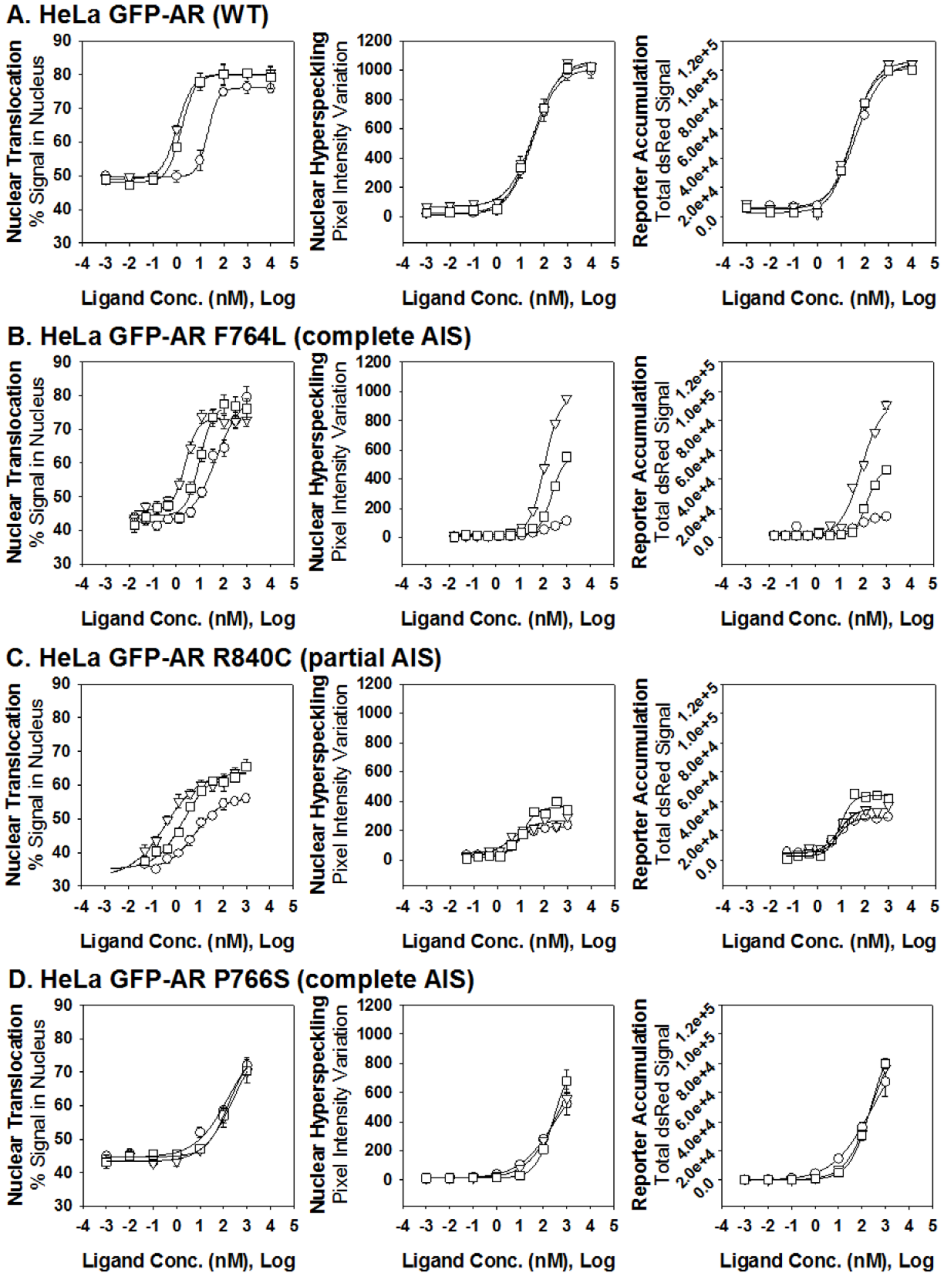
Dose-dependent effects of three AR agonists on AR nuclear translocation, nuclear hyperspeckling, and transcriptional reporter gene activity. HeLa cells were stably transfected with wtAR (A), AR F764L (B), AR-R840C (C) and AR-P766S (D) and transiently transfected with the reporter pARR_2_PB-dsRED2skl. Cells were maintained in 5% SD-FBS media for 12 hr and treated with the indicated compounds for 18 hr in 5%SD-FBS. Tested compounds given at logarithmic concentrations (10^−14^ to 10^−5^ M) include the following AR agonist: DHT (-○-),Mibolerone (-▵-), and R1881(-□-).

Emphasizing the specificity and reproducibility of the HCA approach, our previous GFPAR HeLa results [15] were duplicated with all three ligands. As shown in Fig. 4 and Table 1, DHT, R1881, and Mb induced a significant increase in the percent of the GFP signal in the nucleus (by approximately 30%) with nM EC50 concentrations (DHT - 10.5 nM, R1881 - 0.8 nM, Mb - 1.1 nM). All three agonists induced significant hyperspeckling compared to vehicle control by approximately 50-fold, at EC50 concentrations that, in agreement with previous data [35], were significantly higher than for nuclear translocation (DHT: 29.1 nM; R1881: 28.5 nM; and, Mb: 29.4 nM). Induction of hyperspeckling by the three ligands was tightly correlated to AR transcriptional activity, with similarly high increases in reporter accumulation (an average of 100-fold induction for each ligand) at similar EC50 concentrations as observed for the hyperspeckling response (DHT: 39.2 nM; R1881: 30.1 nM; and, Mb; 31.6 nM).

**Table 1:** Calculated Maximal and EC50 Responses of AIS Mutations in HeLa Cells.

Response	Untreated	DHT_Max_	DHT_EC50_	R1881_Max_	R1881_EC50_	Mb_Max_	Mb_EC50_
HeLa Wild Type							
* Percent Signal in Nucleus*	49.0±4.5	75.6±2.1	10.5±1.9	81.2±1.9	0.8±0.5	80.5±1.9	1.1±0.7
* Hyperspeckling*	20.5±10.1	950±43	29.1±4.1	1138±66	28.5±4.1	1101±61	29.4±5.2
* Transcriptional Activity*	1.0±4	105±2	39.2±5.1	112±1.5	30.1±4.4	113±1.4	31.6±3.9
HeLa F764L							
* Nuclear Translocation*	42.2±0.9	80.3±1.0	49.6±8.4	77.1±1.3	10.0±1.6	73.2±0.8	2.4±0.3
* Hyperspeckling*	7.3±1.8	118±3	142±17	561±10	233±16	965±8	114±4
* Transcriptional Activity*	2.6±0.7	14.5±0.9	72.6±25.9	46.4±0.5	150±7	92.9±2.2	87.5±11.2
HeLa R840C							
* Nuclear Translocation*	35.2±0.8	56.2±0.8	6.7±1.4	64.1±0.9	2.2±0.12	63.7±1.1	0.37±0.12
* Hyperspeckling*	32.6±7.3	222±7.0	7.6±1.3	361±20	11.7±2.7	269±20	4.3±2.0
* Transcriptional Activity*	3.2±.74	28.5±0.8	7.8±1.0	44.0±1.7	9.1±1.5	35.3±1.4	8.0±1.6
HeLa P766S							
* Nuclear Translocation*	43.2±1.2	79.2±2.1	151±12	76.5±1.9	165±15	78.9±1.5	165±15
* Hyperspeckling*	12.6±2.3	752±25	251±33	801±38	283±21	785±30	264±10
* Transcriptional Activity*	1.1±3.1	95±2.3	243±9	101±2	268±19	98±2.2	245±15

Values calculated by applying the 4-parameter curve fit algorithm available in SigmaPlot to multiple replicate experiments and represent Mean±S.E.M. All data are presented as absolute numerical measurements.

Calculated maximal responses from AR HCA. EC50 values are presented for Nuclear Translocation, Hyperspeckling and Transcriptional Activity after stimulation with three AR agonists (DHT, Mibolerone and R1881) in HeLa transfected with wild type AR, AR-F764L, AR-R840C and AR-P766S. EC50 represents the nM concentration of agonist that provokes a response halfway between the baseline and maximal induction. Maximal response achieved with each agonist for each HTM parameter is expressed as % signal present in the nucleus for Nuclear Translocation, as fold-induction from time point 0 for Hyperspeckling and Transcriptional Activity.

#### GSF cells from normal individuals

**Figure 5:**
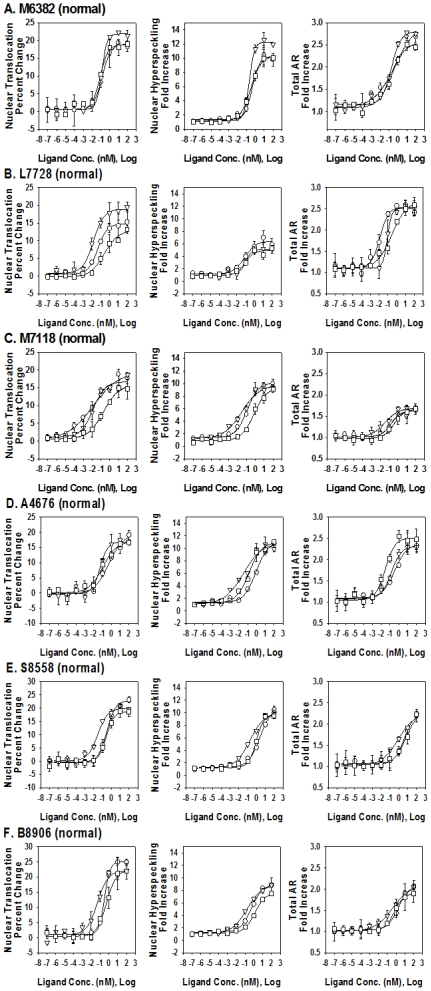
Dose dependent effects of three AR agonists on AR nuclear translocation, nuclear hyperspeckling and total AR. Data of fold-increase are presented from 6 genital skin fibroblasts cell lines obtained form normal individuals at the time of circumcision. Cells were maintained in 5% SD-FBS media for 12 hr and treated with the indicated compound for 18 hr in 5%SD-FBS. Tested compounds given at logarithmic concentrations (10^−14^ to 10^−5^ M) include the following AR agonist: DHT (-○-), Mibolerone (-▵-), and R1881(-□-).

To directly visualize endogenous AR expression in GSF cell lines, our standard GFP-based approach was modified to include anti-AR immunofluorescence (Fig. 1A, 5, and Table 2). To better facilitate direct comparisons between the GSF cell lines, results were normalized to percent change or fold-change from vehicle-treated controls. When treated with the standard panel of AR agonists, the six normal GSF cell lines (M6382, L7728, M7118, A4676, S8558, B8906) all demonstrated a dose-dependent increase in the percent of the AR signal found in the nucleus. The average percent increase with DHT, R1881 and Mibolerone in nuclear translocation was 20.1±3.7%, 16.4±4.0% and 19.4±2.5%, and this was achieved at EC50 concentrations of 0.15±0.14, 0.22±0.16 and 0.046±0.041 nM, respectively (Fig. 5, Table 2). The average increases in nuclear hyperspeckling and total AR signal were 8- and 2-fold, respectively, and this was achieved at subnanomolar EC50 values of the three ligands (Fig. 5, Table 2).

**Table 2:** Calculated Max and EC50 Responses of Normal GSF Cell Lines.

Response	Untreated	DHT_Max_	DHT_EC50_	R1881_Max_	R1881_EC50_	Mb_Max_	Mb_EC50_
GSF M6382 (Normal)							
* Percent Signal in Nucleus*	51.2±3.2	70.1±0.4	0.14±0.01	70.0±0.9	0.080±0.023	73.1±0.5	0.12±0.01
* Hyperspeckling*	7.5±1.2	76.5±2.6	0.45±0.10	77.7±1.6	0.31±0.04	92.2±1.2	0.21±0.02
* Total AR*	74.9±5.4	224±25	0.49±0.58	191±9	0.19±0.10	210±7	0.18±0.06
GSF L7728 (Normal)							
* Nuclear Translocation*	50.2±2.1	65.0±0.5	0.047±0.011	63.1±1.0	0.26±0.13	69.1±0.5	0.015±0.003
* Hyperspeckling*	25.0±3.7	162±10	0.077±0.044	124±5	0.077±0.036	128±6	0.097±0.030
* Total AR*	12.5±3.1	31.8±0.8	0.012±0.004	33.6±1.4	0.28±0.12	31.2±0.5	0.084±0.013
GSF M7118 (Normal)							
* Nuclear Translocation*	31.4±1.9	50.9±1.5	0.013±0.009	47.2±0.8	0.19±0.07	48.1±0.9	0.013±0.007
* Hyperspeckling*	60±7.1	606±18	0.12±0.03	560±27	0.67±0.20	586±35	0.030±0.016
* Total AR*	82±10	133±5	0.16±0.10	136±4	0.36±0.15	138±4	0.033±0.01
GSF A4676 (Normal)							
* Nuclear Translocation*	32.1±2.1	51.6±0.6	0.49±0.13	49.2±1.4	0.11±0.06	48.9±0.6	0.052±0.016
* Hyperspeckling*	43±6.2	469±10	0.84±0.17	473±10	0.14±0.03	464±39	0.032±0.023
* Total AR*	53±14	125±3	0.33±0.14	108±4	0.030±0.010	122±2	0.14±0.04
GSF S8558 (Normal)							
* Nuclear Translocation*	35.2±3.1	58.1±0.4	0.31±0.05	47.2±0.8	0.19±0.07	55.1±0.5	0.028±0.006
* Hyperspeckling*	35±6.6	374.5±8	0.90±0.40	337±6	0.92±0.14	355±7	0.18±0.04
* Total AR*	50±9	111±2	1.0±0.2	114±3	0.95±0.21	114±2	0.85±0.22
GSF B8906 (Normal)							
* Nuclear Translocation*	39.2±0.2	64.3±0.4	0.32±0.03	61.5±0.5	0.53±0.11	61.3±0.7	0.051±0.017
* Hyperspeckling*	45±5.2	405±7	0.53±0.08	343±5	1.3±0.2	401±6	0.17±0.03
* Total AR*	59±5	123±1	1.7±0.3	112±1	0.63±0.08	128±6	0.38±0.09

Average Normal Response		DHT_Change_	DHT_EC50_	R1881_Change_	R1881_EC50_	Mb_Change_	Mb_EC50_
* Nuclear Translocation (% Point change)*		20.1±1.4	0.15±0.06	16.5±1.6	0.23±0.07	19.4±1.0	0.046±0.016
* Hyperspeckling (fold change)*		9.6±0.7	0.49±0.14	8.8±0.9	0.57±0.20	9.5±1.0	0.12±0.04
* Total AR (fold change)*		2.3±0.2	0.61±0.26	2.2±0.2	0.44±0.15	2.3±0.2	0.28±0.12

Values calculated by applying the 4-parameter curve fit algorithm available in SigmaPlot to multiple replicate experiments. For individual cell lines, all data are presented as absolute numerical measurements. For the average normal response, nuclear translocation is reported as the percentage point change from untreated samples whereas hyperspeckling and total AR is reported as fold change from untreated samples.

Calculated maximal responses from AR HCA. EC50 values for Nuclear Translocation, Hyperspeckling and Total AR increase after stimulation with three AR agonists (DHT, Mibolerone and R1881) in GSF from 6 normal individuals (M6382, L7728, M7118, A4676, S8558, B8906). EC50 represents the nM concentration of agonist that provokes a response halfway between the baseline and maximal induction. Maximal response achieved with each agonist for each HTM parameter is expressed as % signal present in the nucleus for Nuclear Translocation, as fold-induction from time point 0 for Hyperspeckling and Total AR content.

### Image Based Analysis of AR- F764L Function

#### HeLa

We next applied the same technique to HeLa cells stably expressing the AR-F764L mutation (Fig. 4, Table 1). When cells were treated with the panel of agonists, ARF764L translocated into the nucleus with similar increases in the percent of nuclear GFP signal as wild type receptor (DHT: ↑ 38%; R1881: ↑ 36%; and, Mb: ↑ 31%). However, the responses were observed at higher EC50 concentrations, with the greatest shift for the endogenous ligand DHT (DHT: 49.6 nM; R1881: 10.0 nM; and, Mb: 2.4 nM). Surprisingly, the hyperspeckling response demonstrated a clear distinction between the three compounds; DHT produced a weak hyperspeckling response at elevated ligand concentrations (15-fold increase; EC50: 142 nM), R1881 induced a significantly stronger response at even higher ligand concentrations (77-fold increase; EC50: 233 nM) while Mb was able to induce the highest levels of hyperspeckling (132-fold increase) at an EC50 concentrations of 114 nM. Similarly to wild type AR, transcriptional activity correlated with hyperspeckling. Induction of transcriptional activity was significantly stronger with Mb than R1881 and DHT (DHT: 6-fold; R1881: 18-fold; Mb: 36-fold), and occurred at higher EC50 concentrations than with the wild type receptor (DHT: 102 nM; R1881: 160 nM; and, Mb: 87 nM).

#### GSF

**Figure 6:**
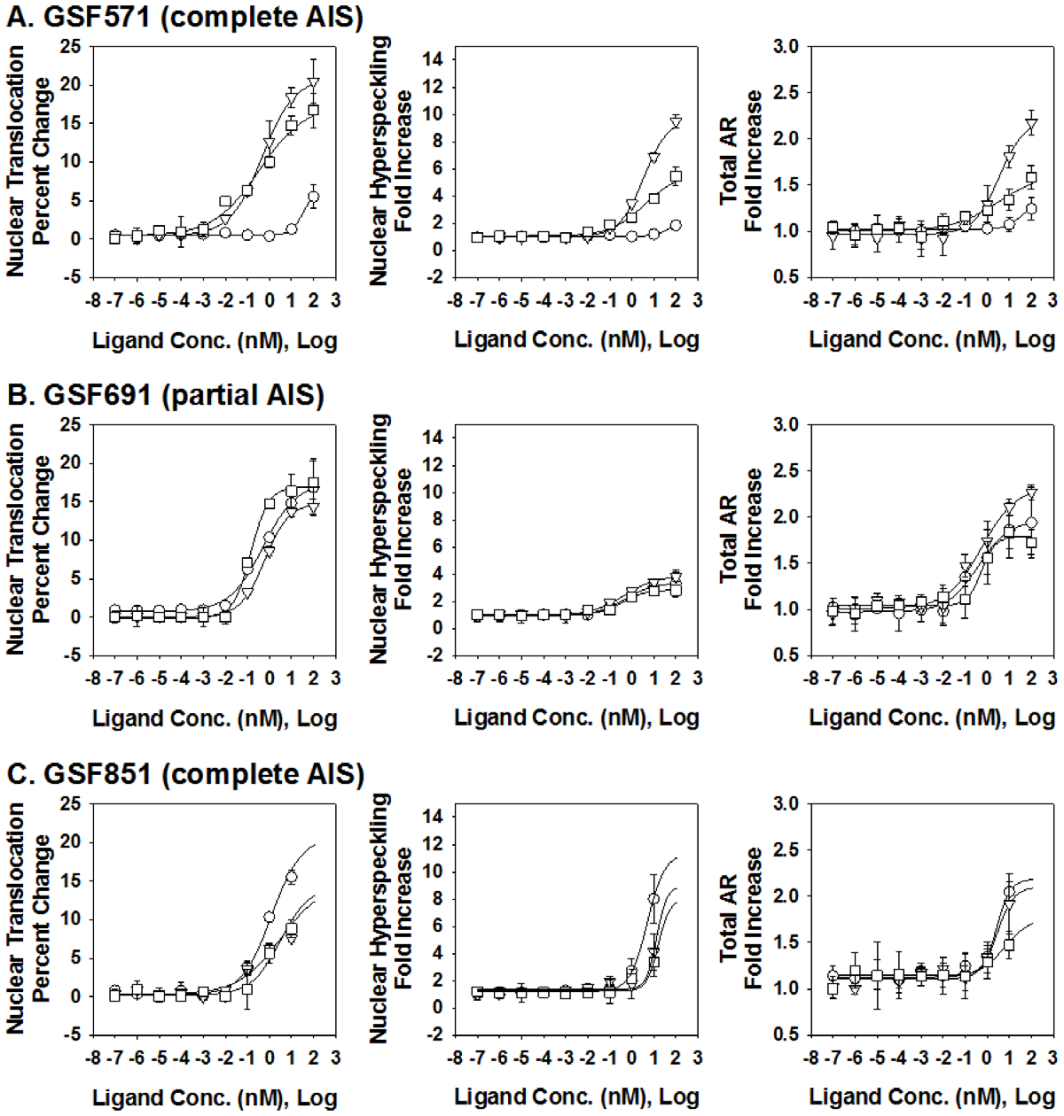
Dose dependent effects of three AR agonists on AR nuclear translocation, nuclear hyperspeckling and total AR fold-increase. Data is presented from genital skin fibroblasts cell lines obtained from AIS patients with AR mutations AR-F764L (GSF571), AR-R840C (GSF840) and AR-P766S (GSF851). Cells were maintained in 5% SD-FBS media for 12 hr and treated with the indicated compound for 18 hr in 5%SD-FBS. Tested compounds given at logarithmic concentrations (10^−14^ to 10^−5^ M) include the following AR agonist: DHT (-○-), Mibolerone (-▵-), and R1881(-□-).

When we examined the nuclear translocation response in GSF cells harvested from a patient harboring the F764L mutation (Figure 6, Table 3), both R1881 and Mb were able to induce a normal response (17.0% and 20.9% increase), whereas the effect of DHT was below the range found in normal samples (6.5% increase). In addition, the minor DHT respon se required significantly higher concentration (EC50 = 35.3 nM) than the agonist-induced responses in normal GSF cell lines. When the hyperspeckling response was examined (Figure 6, Table 3), a similar pattern emerged. Both R1881 and Mb induced strong responses, nearly equivalent to that observed in the normal GSF controls (5.7- and 9.7-fold increase) whereas DHT was weak (1.8-fold increase). Again, the necessary concentration to induce a DHT response was higher (EC50: 83.7 nM) than those needed for the R1881 (EC50: 3.9 nM) or Mb (EC50: 3.5 nM), although all EC50 concentrations were higher than the range observed in the normal GSF control samples. When ligand dependent stabilization of the F764L receptor was examined in GSFs, only R1881 and Mb induced responses within the normal range of wild type GSF cells (e.g., a 1.3- to 2.6-fold increase in total AR signal). These results indicate that GSFs harboring the F764L mutation exhibited reduced responsiveness to the endogenous ligand DHT, and that, across all simultaneously evaluated measurements, a nearly complete pharmacological rescue was possible with synthetic agonists, particularly Mibolerone.

**Table 3:** Calculated Max and EC50 Responses of AIS GSF Cell Lines.

Response	Untreated	DHT_Max_	DHT_EC50_	R1881_Max_	R1881_EC50_	Mb_Max_	Mb_EC50_
GSF 571 (F764L)							
* Nuclear Translocation*	37.2±4.5	43.0±0.1	35.3±4.3	54.2±0.7	0.35±0.13	58.1±0.2	0.47±0.04
* Hyperspeckling*	72.1±7.6	130±4	83.7±18.4	411±16	3.9±1.4	699±9	3.5±0.4
* Total AR*	45.1±2.2	58.8±0.7	33.6±10.0	73.2±2.9	3.2±2.8	99.4±2.0	3.4±0.9
GSF 691 (R840C)							
* Nuclear Translocation*	20.1±1.1	37.6±0.8	0.51±0.14	37.0±0.4	0.14±0.02	34.9±0.3	0.61±0.07
* Hyperspeckling*	15.3±4.1	51.2±1.2	0.47±0.09	43.1±1.5	0.38±0.13	59.2±1.8	0.44±0.12
* Total AR*	40.1±1.9	78.6±2.8	0.39±0.17	71.4±2.1	0.54±0.20	93.0±5.2	0.55±0.31
GSF 851 (P766S)							
* Nuclear Translocation*	30.1±2.1	50.8±0.5	1.2±0.2	44.4±0.6	3.2±1.0	44.9±1.1	3.2±1.9
* Hyperspeckling*	25.1±0.9	286±3	5.1±0.3	201±6	15.7±3.3	224±5	13±2
* Total AR*	135±32	294±27	2.7±0.4	239±5	5.9±2.9	283±5	2.8±0.8

*Values calculated by applying the 4-parameter curve fit algorithm available in SigmaPlot to multiple replicate experiments. All data are presented as absolute numerical measurements.*

Calculated maximal responses from AR HCA.

EC50 values for Nuclear Translocation, Hyperspeckling and Total AR increase after stimulation with three AR agonists (DHT, Mibolerone and R1881) in GSF from AIS patients with AR mutations F764L, R840C and P766S. EC50 represents the nM concentration of agonist that provokes a response halfway between the baseline and maximal induction. Maximal response achieved with each agonist for each HTM parameter is expressed as % signal present in the nucleus for Nuclear Translocation, as fold-induction from time point 0 for Hyperspeckling and Total AR content.

### Image Based Analysis of AR- R840C Function

#### HeLa

When HeLa cells stably expressing the R840C PAIS mutation were treated with the panel of agonists (Fig. 4, Table 1), they also demonstrated the ability to achieve increases in nuclear translocation similar to the wild type receptor (DHT: ↑ 21%; R1881: ↑ 29%; and, Mb: ↑ 29%), and at similar EC50 concentrations (DHT: 6.7 nM; R1881: 2.2 nM; and, Mb: 0.37 nM). However, because the receptor is significantly more cytoplasmic in the untreated state, the relative maximal nuclear translocation appears reduced (Fig. 4, Table 1). There was reduced ability to induce hyperspeckling (DHT: 7-fold increase; R1881: 11-fold increase; and, Mb: 8-fold increase), however, responses occurred at lower EC50 concentrations compared to the wild type receptor (DHT: 7.6 nM; R1881: 11.7 nM; and, Mb: 4.3 nM). Transcriptional activation mirrored the hyperspeckling results, with only minor increases (DHT: 9-fold; R1881: 14-fold; Mb: 11-fold) at EC50 concentrations below those observed with the wild type receptor (DHT: 7.8 nM; R1881: 9.1 nM; and, Mb: 8.0 nM).

#### GSF

All three compounds tested induced significant nuclear translocation responses (14.8% to 17.5% increase), in the range observed in normal GSFs, at EC50 of 0.14-0.61 nM (Fig. 6, Table 3). In contrast, only a moderate response in hyperspeckling was observed (2.8- to 3.9-fold increase), at normal EC50 concentrations (EC50: 0.38–0.47 nM). Ligand-dependent stabilization of the receptor occurred with all three compounds in a way that was similar to normal GSF samples (1.8- to 2.3-fold; EC50: 0.17–0.55 nM). In general, in this cell line the results indicate that agonist binding to AR-R840C generates normal nuclear translocation and receptor stabilization, but significantly reduced ability of AR to establish subnuclear hyperspeckling [15].

### Image Based Analysis of AR-P766S Function

#### HeLa

All three agonists induced significant nuclear translocation, hyperspeckling and transcriptional activation of AR-P766S to wild-type AR levels, however, these responses were observed at significantly higher concentrations of ligand (Fig. 4, Table 1). In contrast to the F764L mutation, differential ligand selectivity was absent in AR-P766S.

#### GSF

All three agonists induced significant nuclear translocation (14.3%–20.7%) when used at higher concentrations (EC50: 1.2–3.2 nM) in GSFs harboring the AR-P766S mutation (Fig. 6, Table 3). This was associated with marked hyperspeckling responses (8.0- to 11.4-fold increase; EC50: 5.1–15.7 nM), and receptor stabilization (e.g., 1.8- to 2.2-fold increase; EC50: 2.7–5.9 nM). Overall, GSF AR-P766S was similar to GSF AR-F764L. It showed reduced ligand sensitivity, although responsiveness was complete, and not ligand-specific.

## Discussion

We hypothesized that AR point mutations localized in the ligand binding domain and associated with normal ^3^H-DHT B_max_ and K*_d_* may be reversible, and proved this by using the new AR HCA technology and conventional assays, such as NC-TDI. HCA not only proved to be a powerful tool to understand the functional abnormalities of AR mutations directly in patient-derived specimens, but was also utilized to predict clinical responses to a variety of experimental treatments.

### NC-TDI

Experiments were performed to establish if the three mutants were affected by an impairment of the NH_2_-COOH terminal interaction, and if so, if this impairment would be amenable to normalization using conditions that enhance stability of binding, for instance by increasing concentrations of natural or synthetic AR agonists. NC-TDI was impaired in the two mutations associated with increased ligand-receptor dissociation rate; AR-F764L and AR-P766S. ARF764L demonstrated a ligand-dependent partial rescue of NC-TDI to approximately 50% of wild type, at high concentrations (e.g.: 200 nM) of Mibolerone, but not of R1881 and DHT. AR-P766S demonstrated a complete rescue of NC-TDI with all four ligands, in a way that was proportional to the concentration used, and reached complete normalization at 200 nM. These data confirmed that notion that abnormal NC-TDI is a hallmark of increased ligand-receptor dissociation rate [36], and suggested that a stabilization of ligand-receptor interaction occurred at higher concentrations of ligand. That NC-TDI recovery was only partial and ligand-dependent in F764L was surprising. This observation may be related to the fact that F764 makes direct ligand contact [37], and its replacement by ligand may alter the conformation of the ligand binding pocket which results in increased affinity for ligands other than DHT. The R840C mutation demonstrated increased NC-TDI's with all ligands and concentrations tested compared to wild type. We knew from the outset that AR-R840C affected ^3^H-DHT binding causing thermolability, not increased ligand-receptor dissociation rate. Thus, it should not have been a surprise to find that NC-TDI was unaffected in AR-R840C. The mechanism of androgen insensitivity in the context of an AR mutation showing increased NC-TDI is unclear. Because AF2 is important not only for NC-TDI, but also for p-160 coactivators recruitment [38], it could be speculated that the increased interaction between AF2 and the N-terminus observed in AR-R840C may be associated with improper recruitment of p-160 coactivators, and thus decreased AR transcriptional activity. Finally, as the transient expression of the wt and mutant AR fusions in the N-C assay precludes measurement of absolute AR levels per cell, we cannot dismiss the possibility that the observed increase in N-C interactions may be influenced by stabilization of the LBD by a type, or concentration, of ligand. Given the preponderance of our other data, this less interesting possibility seems remote.

### HCA Experiments in Hela Transfected with Wild Type AR vs. GSF from Normally Virilized Individuals

HCA activities measured in primary GSF cultures from six normal patients that were used as a control were within a relatively small interval. These minor differences probably reflect a unique intracellular equilibrium of molecules regulating AR activation, or polymorphisms of the polyglutamine repeat of AR exon 1. It is also possible that the different HCA activities observed in these cell lines reflect different degrees of normal virilization that are present in the general population.

A number of reproducible, cell line-dependent differences were detected in the control groups between HeLa and GSF. **First**, as previously described [15], EC50 for ligand-dependent nuclear translocation were higher compared with hyperspeckling and transcriptional activity in HeLa-AR cells using R1881 and Mibolerone (by a factor of approximately 30-fold), or DHT (by a factor of approximately 3-fold). In contrast no such difference was present in GSF cells. **Second**, the EC50's for hyperspeckling were lower in GSF compared with HeLa by a factor of approximately 70-fold. **Third**, the fold-induction of hyperspeckling (a surrogate of transcriptional activity) was lower by a factor of 5-fold in GSF cells compared to HeLa-AR. The reasons for these discrepancies are not clear. The first discrepancy may be related to decreased ability of ligand-bound AR to interact with the machinery necessary for nuclear import in HeLa cells, or to the presence of a competing androgen-binding protein in the cytosol but not the nucleus of HeLa, so that higher concentrations of androgen would be necessary to complete functions that take place in the cytoplasm, such as nuclear import, but not in the nucleus, such as hyperspeckling or transcriptional activity. The third discrepancy may be related to decreased transcriptional activity in GSF reported by others [39], or to the preponderance in these cell lines of AR co-repressors [40].

### AR-F764L

These studies have shown that the recovery of AR-F764 activity (e.g.: 45% of control) occurs in a Mibolerone-dependent way. Similar results were obtained in GSF containing ARF764L or transfected HeLa; the main difference being that Mibolerone was more effective in GSF, with a recovery of hyperspeckling, a surrogate marker of transcription, at 100% of control levels. These studies reconcile the CAIS phenotype of this patient with the fact that at the critical time of genital differentiation the fetus was exposed to the endogenous ligand DHT, which is inactive with AR-F764L. A Mibolerone-specific recovery of function argues that this agonist fits inside the mutated ligand-binding pocket of AR-F764L more properly than the physiologic ligand DHT. Stabilization of the receptor-ligand complex with high doses of Mibolerone rescued NC-TDI to 50% of control, and this was associated by improved ligand retention in the ligand-binding pocket of the receptor, and by an increase in transcription.

### AR-P766S

We observed complete recovery of AR NC-TDI and HCA functions with pharmacologic concentrations of DHT, R1881 and Mibolerone for mutant AR-P766S. Because the interaction between ligands and AR-P766S was highly unstable at physiologic concentrations, NC-TDI and transcription were compromised to the point that the patient developed a CAIS phenotype. Supraphysiologic concentrations of the three ligands improved ligand-receptor stability, and normalized NC-TDI and transcriptional activity to control levels.

### AR-R840C

HCA analysis in HeLa and GSF cells showed that nuclear translocation of AR-R840C could be almost normalized at higher concentration of each ligand, however neither hyperspeckling nor transcriptional activity were recovered. Because this mutation was not associated with instability of receptor-ligand binding, lack of response to experimental conditions coupled with increased receptor-ligand stability was not surprising. As stated above, the presence of increased NC-TDI may be associated with decreased recruitment of coactivators and decreased transcriptional activity.

In conclusion these studies have generated several new concepts. **First**, AIS mutations with normal K*_d_*, B_max_, and increased ligand dissociation rate can be rescued. In contrast, other mutations with a slightly different phenotype (i.e. normal K*_d_*, B_max_, ligand dissociation rate, but abnormal thermostability) were not rescued. Thus, to this point the biochemical signature of AR mutations that can be rescued consists in normal K*_d_*, B_max_, and increased ligand dissociation rate. In support of these observations, the PAIS patients who responded to treatment in the paper of Grino [6], [10] had an AR mutation with this biochemical signature. **Second**: mutations with normal K*_d_* and B_max_ and increased ligand dissociation rate are associated with decreased NC-TDI. NC-TDI is a surrogate of ligand-receptor binding stability, thus such mutations can be rescued by using conditions that increase ligand-receptor binding stability, such as use of pharmacologic doses of natural or synthetic ligands. **Third**: These studies prove the fact that HCA can generate in a short period of time useful information to direct personalized medicine for patients affected by AIS. The extent that these studies can be generalized will be determined by AR HCA studies of additional patients with similar phenotypes.

### How AR HCA Data Could Be Used in a Clinical Setting

More studies are needed with additional cell lines before direct translation into clinical practice. Safety will be an important issue, particularly for the synthetic agonists, which are not FDA-approved. The CAIS phenotype of patients AR-P766S and AR-F764L argues against treating these patients, because 100% of them reared as females maintained throughout life a female gender identity [41]. Nevertheless, the prediction is that significant virilization may have occurred after observing the in vitro response of AR-P766S and AR-F764L. PAIS patients would clearly be the first beneficiaries of this technology. Whether the small improvement in AR activity observed at pharmacologic doses of agonists for AR-R840C would have resulted in an improvement of the PAIS phenotype of this patient remains unknown; however, experience with similarly undervirilized individuals argues that even small responses improve the outcome of surgical repairs [6].

In conclusion, this work represents a step forward toward the utilization of modern high-speed microscopic techniques for the diagnosis and treatment of diseases related to AR. It will be interesting to expand these studies to include evaluation of more global effects of AR rescue using different concentrations of ligand, or with ligands other than the endogenous AR agonists. Future investigation will continue to analyze AR malfunction in AIS and other clinical conditions, such as prostate cancer, hypospadias, cryptorchidism and sarcopenia.
